# Notch stimulates growth by direct regulation of genes involved in the control of glycolysis and the tricarboxylic acid cycle

**DOI:** 10.1098/rsob.150155

**Published:** 2016-02-17

**Authors:** Vera Slaninova, Michaela Krafcikova, Raquel Perez-Gomez, Pavel Steffal, Lukas Trantirek, Sarah J. Bray, Alena Krejci

**Affiliations:** 1 Faculty of Science, University of South Bohemia, Branisovska 31, 37005 Ceske Budejovice, Czech Republic; 2 Institute of Entomology, Biology Centre, Czech Academy of Sciences, 37005 Ceske Budejovice, Czech Republic; 3 Central European Institute of Technology, Masaryk University, Kamenice 5, 625 00 Brno, Czech Republic; 4 Department of Physiology, Development and Neuroscience, University of Cambridge, Downing Street, Cambridge CB2 3DY, UK

**Keywords:** metabolism, Notch targets, Warburg effect, glycolytic shift, tissue growth

## Abstract

Glycolytic shift is a characteristic feature of rapidly proliferating cells, such as cells during development and during immune response or cancer cells, as well as of stem cells. It results in increased glycolysis uncoupled from mitochondrial respiration, also known as the Warburg effect. Notch signalling is active in contexts where cells undergo glycolytic shift. We decided to test whether metabolic genes are direct transcriptional targets of Notch signalling and whether upregulation of metabolic genes can help Notch to induce tissue growth under physiological conditions and in conditions of Notch-induced hyperplasia. We show that genes mediating cellular metabolic changes towards the Warburg effect are direct transcriptional targets of Notch signalling. They include genes encoding proteins involved in glucose uptake, glycolysis, lactate to pyruvate conversion and repression of the tricarboxylic acid cycle. The direct transcriptional upregulation of metabolic genes is PI3K/Akt independent and occurs not only in cells with overactivated Notch but also in cells with endogenous levels of Notch signalling and *in vivo*. Even a short pulse of Notch activity is able to elicit long-lasting metabolic changes resembling the Warburg effect. Loss of Notch signalling in *Drosophila* wing discs as well as in human microvascular cells leads to downregulation of glycolytic genes. Notch-driven tissue overgrowth can be rescued by downregulation of genes for glucose metabolism. Notch activity is able to support growth of wing during nutrient-deprivation conditions, independent of the growth of the rest of the body. Notch is active in situations that involve metabolic reprogramming, and the direct regulation of metabolic genes may be a common mechanism that helps Notch to exert its effects in target tissues.

## 1. Background

Cellular metabolism is considered as one of the key factors regulating cell survival, proliferation, transcription, chromatin status and cell signalling [1,2]. Specific modes of metabolism seem to be crucial for individual cell types and their disruption can have profound consequences for cell function and survival [3]. Rapidly proliferating cells, including cancer cells, have to alter their metabolism in order to satisfy their high demands for compounds needed for fast cell growth and divisions. This involves a metabolic switch towards high glycolysis uncoupled from the tricarboxylic acid cycle (TCA) and oxidative phosphorylation, known as the Warburg effect [4,5]. There are precedents for glycolytic shift in many proliferating as well as in some non-proliferating cell types, including lymphoblasts [6], endothelial cells during angiogenesis [7] and stem cells [8]. Moreover, during *Drosophila* development, glycolytic shift occurs in mid-embryogenesis and lasts until late larval stages [9,10].

Given the importance of metabolic parameters to cell homoeostasis, it is not surprising that several signalling pathways are known to regulate the cellular metabolic profile. For example, signalling through growth factors or insulin receptors is known to trigger the PI3K/Akt pathway that in turn enhances glucose uptake, glycolysis and lipid synthesis [11]. Similarly, several signalling pathways activate the transcription factor HIF-1, which promotes a metabolic switch towards the Warburg effect under both normoxic and hypoxic conditions [12]. Furthermore, the mTORC1 complex responds to changes in intracellular ATP/ADP, amino acid levels plus systemic nutrients to alter the levels of protein translation, glycolysis and lipid synthesis in a manner that promotes anabolic cell growth and proliferation [13]. An increasing appreciation of how cancer cells can often hijack such signalling mechanisms in order to initiate metabolic reprogramming has therefore emerged as a central theme in contemporary cancer treatment [14].

The Notch signalling pathway regulates cell fate determination during development and it is also known to promote cell growth and division [15]. It can function as both a tumour suppressor and a tumour-promoting factor in several types of haematopoietic cancers and solid tumours [16]. During ligand-stimulated activation, the plasma membrane-localized Notch receptor is cleaved liberating its intracellular domain (N^icd^). N^icd^ then translocates into the nucleus where it binds to the transcription factor from the CSL family thus converting it into a transcriptional activator. Recent evidence has suggested a functional link between Notch signalling and cellular metabolic status. For example, metabolic genes are upregulated in Notch-dependent T-cell lymphoblastic leukaemia [17,18] or breast cancer cells [19]. The MCF7 breast cancer cell line engineered to hyperactivate Notch undergoes glycolytic switch that is dependent on the PI3/Akt signalling [19]. The Notch pathway has also been reported to collaborate with the metabolically regulated HIF-1 to promote cell survival and invasiveness [20,21], and perturbed Notch signalling was shown to cause defects in mitochondrial metabolism [22]. On the other hand, there is evidence of a feedback relationship where disturbed cell metabolism affects the levels of Notch signalling [23]. Whether metabolic genes are direct targets of the Notch pathway and whether this regulation happens under non-pathological conditions such as during normal development is not clear.

In this paper, we report that several key metabolic regulator genes are direct transcriptional targets of the Notch pathway, mediating a cellular metabolic shift towards the Warburg effect*.* This regulation happens after a short pulse of Notch activity both in cells overexpressing Notch receptor as well as in cells with endogenous levels of Notch signalling and *in vivo*. Loss of Notch signalling results in lower expression of metabolic genes together with lower growth of the tissue. Consequently, we propose a model in which activated Notch signalling can promote glycolytic shift in target cells, as characterized in tissues during development, during immune response, and in stem cells, as well as in Notch-dependent cancers.

## 2. Results

### 2.1. Several genes involved in the regulation of metabolism contain Su(H) binding sites in their regulatory regions

**Figure 1.**
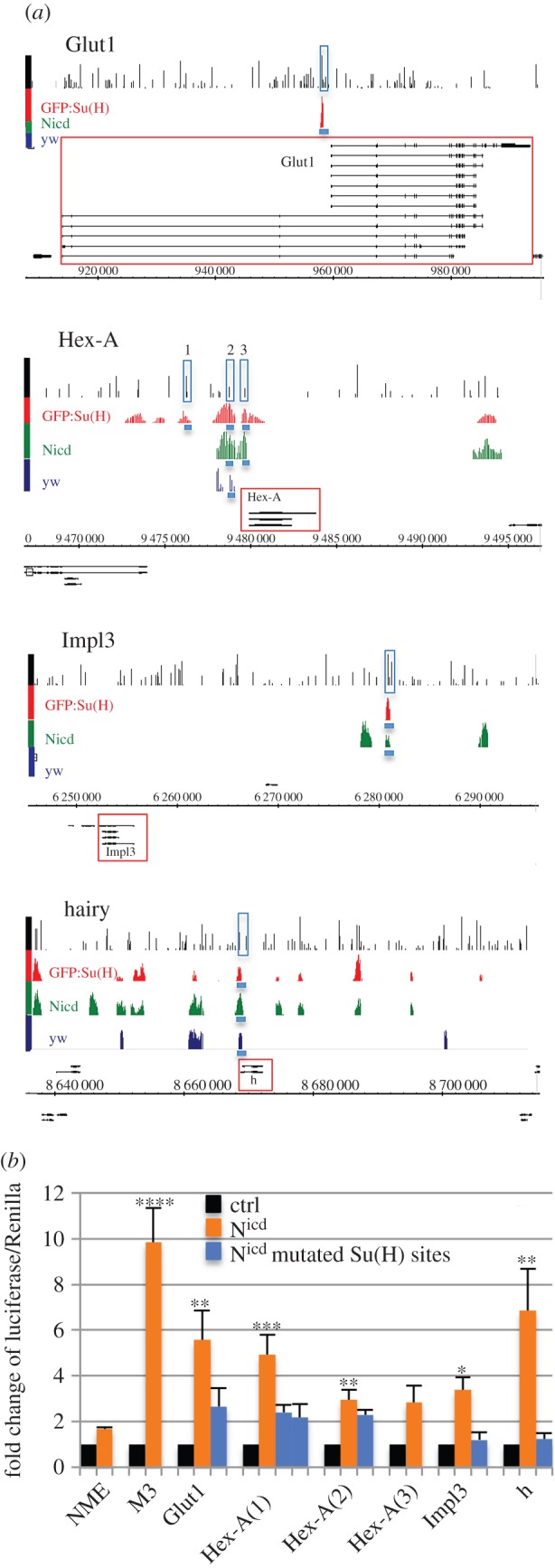
(*Opposite.*) The binding profile of Su(H) at regulatory regions of metabolism-related genes. (*a*) Genomic regions of *Glut1, Hex-A, Impl3* and *hairy* show Su(H) ChIP peaks (enrichment relative to input, log2 scale 0–4) in wing discs from yw (dark blue), ptc-Gal4 > Nicd (green) and ptc-Gal4 > GFP:Su(H) discs (red) [24]. Black lines in first rows indicate computationally predicted Su(H) binding sites based on Patser Su(H) matrix. Gene models are depicted in black, horizontal numbering indicates genomic coordinates according to release 5 of *Drosophila* genome (BDGP R5.12/dm3). Genomic regions cloned for luciferase assays are indicated by blue rectangles. (*b*) Luciferase assay in S2 cells using genomic regions indicated in (*a*). NME is negative control (Notch mutant enhancer); M3 contains Notch responsive element from *bHLHm3.* Blue columns indicate responses of the same regions where *Su(H)* sites were mutated. Two different *Su(H)* sites were mutated in Hex-A region 1. Significance is shown according to Kruskal–Wallis test, comparing groups against NME.

Suppressor of hairless (Su(H)) is the *Drosophila* transcription factor from the CSL family that mediates the Notch response on target gene enhancers. In a previous study, we identified the directly regulated Notch targets, by performing ChIP with *α*-Su(H) antibody in wild-type *yw* wing discs and in discs where either N^icd^ or GFP:Su(H) was overexpressed causing epithelial hyperplasia [24]. To assess whether Notch activity might regulate metabolism-related genes, we searched for Su(H) ChIP peaks in the vicinity of genes involved in metabolism. Several candidates emerged including glucose transporter 1 (*Glut1*), the key enzyme of glycolysis hexokinase A (*Hex-A*) and the glycolysis rate-limiting enzyme lactate dehydrogenase (*Ecdysone-inducible gene L3*, *Impl3*) (figure 1*a*). We selected these genes for further analysis. We also included the transcription factor *hairy* because it showed several Su(H) peaks in its potential regulatory regions and it was previously described as a master regulator of cellular metabolism during hypoxia [25]. We wanted to test the hypothesis that these candidate genes are transcriptionally regulated by Notch and that they can mediate Notch-dependent metabolic changes in target tissues.

To assess if the selected genomic regions respond to Notch activation, we tested their activity in a luciferase assay. In cases where there were more than one Su(H) ChIP peak in the vicinity of a gene, we decided to test those of them that were closer to the 5′ or 3′ end of the gene, that were common to more than one tissue and that had a computationally predicted Su(H) binding site matching the middle of the Su(H) peak. The genomic regions near *Glut1*, *Hex-A*, *Impl3* and *hairy* showed positive responses to N^icd^ that were lost after specific Su(H) binding sites were mutated (figure 1*b*). A region of *Hex-A* closer to the promoter (region 2) was also significantly upregulated by N^icd^, but the predicted Su(H) site we mutated did not abolish this response. Taken together, these results confirmed that there are functional Notch responsive enhancer elements associated with several genes involved in the regulation of cellular metabolism.

### 2.2. Short pulse of Notch activation elicits transcriptional response of *Glut1, Hex-A, Impl3* and *hairy* that is primary and independent of levels of Notch receptor

**Figure 2.**
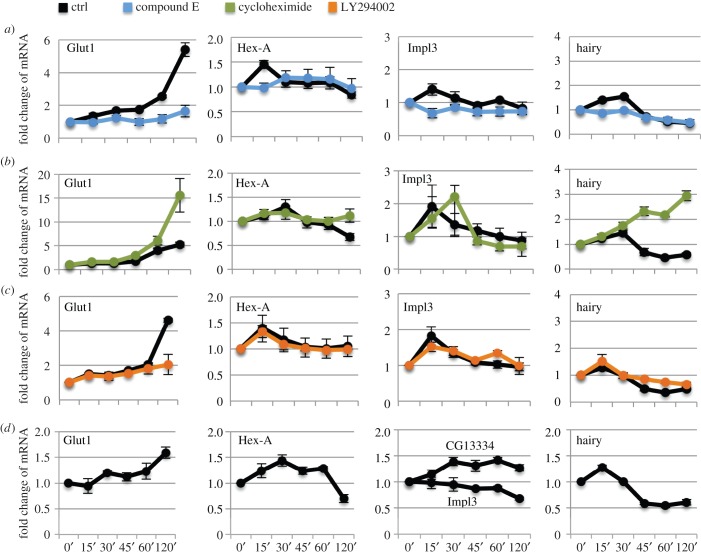
Short pulse of Notch directly activates *Glut1, Hex-A, Impl3* and *hairy* mRNA expression in S2N and DmD8 cells. (*a*–*c*) Notch pathway was activated in S2N cells by EDTA for 15 min and mRNA extracted at indicated time points (minutes, axis labels same for all graphs). Response of control cells is shown in black. Compound E was used to block *γ*-secretase (blue, *a*), cycloheximide blocks protein synthesis (green, *b*) and LY294002 inhibits PI3K/Akt (orange, *c*). (*d*) Notch pathway activated in DmD8 cells by EDTA for 15 min and mRNA extracted at indicated time points (minutes). Data in *a*–*d* are normalized to *CG11306* housekeeping gene. Averages of three biological replicate experiments; error bars are standard errors of the mean. Note that each control in drug treated experiments is an independent set of biological triplicates. Control values from all experiments were analysed together by Willcoxon test to find significant upregulation of *Hex-A*, *Impl3*, *Glut1* and *hairy* at the 15 min time point (*p* < 0.005).

We decided to investigate whether the *Glut1*, *Hex-A*, *Impl3* and *hairy* regulatory elements could regulate Notch-dependent transcription of the endogenous genes in their imminent vicinity. To answer this question, we turned to S2N cells in which we can activate the Notch pathway in a precisely controlled manner using a pulse of EDTA treatment [26]. We activated the Notch pathway for 15 min and then assayed relevant gene mRNA expression in a time course up to 120 min after the activation (figure 2). Enhanced *Hex-A, Impl3* and *hairy* transcription was observed at 15–30 min after Notch activation, after which the response was quenched. *Glut1* mRNA was also upregulated but only 45–60 min after Notch activation. To confirm that responses in S2N cells were specifically dependent on Notch cleavage, we incubated cells with the *γ*-secretase inhibitor compound E alongside the EDTA treatment (figure 2*a*). All four genes lost their transcriptional response, suggesting that, indeed, it is the Notch pathway that is responsible for their upregulation.

To see if Notch-dependent activation of metabolic genes is direct and does not require a Notch-induced secondary protein intermediate, we incubated cells with cycloheximide, to inhibit de novo protein synthesis (figure 2*b*). The response of all genes remained undiminished and in fact the responses of *Glut1* and *hairy* were even more pronounced, suggesting a Notch-dependent transcriptional repressor might be responsible for quenching the initial peak of activation.

Another possible mechanism to explain the increased expression of several metabolic genes is that PI3K/Akt signalling is stimulated following Notch activation, as was observed after hyperactivation of Notch1 in breast cancer cells [19]. To test if the transcriptional upregulation of our candidate genes required PI3K/Akt signalling, we incubated S2N cells with the PI3K inhibitor LY294002 (figure 2*c*). The responses of *Hex-A, Impl3* and *hairy* were unaffected, implying that these genes do not require additional PI3K/Akt signal to respond to Notch activation. In contrast, the *Glut1* response was lowered, although not abolished, upon PI3K/Akt inhibition, suggesting that it may be regulated by combined actions of Notch and PI3K/Akt pathways. Nevertheless, taken together, our experiments with cycloheximide and PI3K/Akt inhibitors support the conclusion that *Glut1, Hex-A, Impl3* and *hairy* are direct transcriptional targets of Notch signalling.

To exclude the possibility that this regulation of metabolic genes was only possible owing to the relatively strong Notch signals we generated when overexpressing the Notch receptor in our S2N cell model, we assayed the Notch responsiveness of the metabolic genes in DmD8 muscle precursor cells. In this case, only the endogenous Notch is present, resulting in much lower receptor levels (see electronic supplementary material, figure S1C, comparing the levels of expression). As shown in figure 2*d, Glut1*, *Hex-A* and *hairy* were also transcriptionally upregulated in DmD8 cells. Although *Impl3* did not respond in this cell type, another lactate dehydrogenase, *CG13334*, that could functionally substitute for *Impl3* was upregulated. *CG13334* is also proximal to two regions with robust Su(H) binding in ChIP in DmD8 cells and in wing discs that were N^icd^ responsive in luciferase assays (see electronic supplementary material, figure S1A and S1B). It is not expressed in S2N cells. These data from DmD8 cells support the hypothesis that Notch-dependent transcriptional upregulation of metabolic genes occurs with endogenous levels of Notch.

### 2.3. Notch activation leads to changes in cellular metabolism

**Figure 3.**
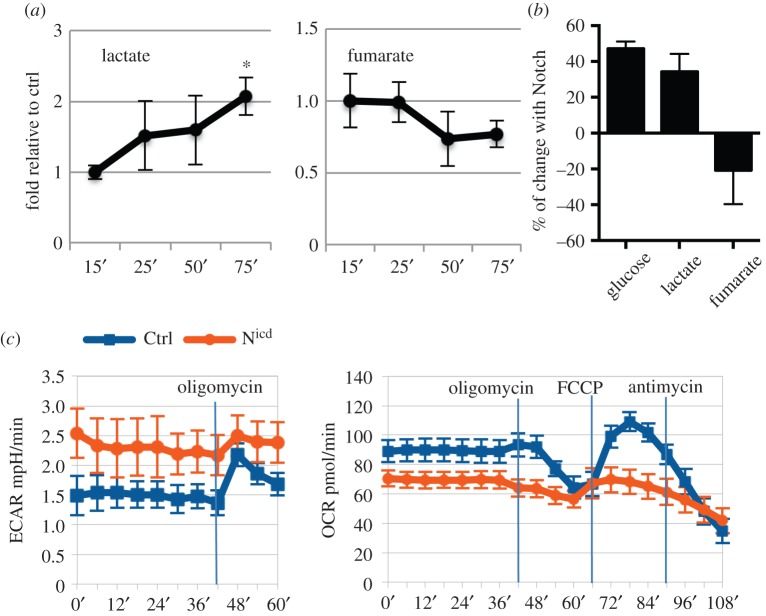
Notch activation mediates changes in cellular metabolism. (*a*) Notch was activated in S2N cells for 15 min after which fresh Schneider medium was added and lactate and fumarate levels were measured at indicated time points by NMR, and n Normalized to metabolite levels in S2N cells without Notch expression (without CuSO_4_ induction) but treated with EDTA to prevent any EDTA non-specific effects. (*b*) Notch was activated in S2N cells for 15 min after which fresh Schneider medium supplemented with isotopically labelled [2–^13^C] glucose was added and glucose, lactate and fumarate levels were measured at the 75 min time point by NMR. Change relative to metabolite levels in S2N cells without Notch expression but treated with EDTA was plotted. (*c*) Extracellular acidification rate (ECAR) and oxygen consumption rate (OCR) of wing discs with N^icd^-induced hyperplasia (abxUbxFLPase; Act > y > Gal4, UAS GFP; FRT82B tubGal80x UAS-Nicd, FRT82B, orange) or in controls (abxUbxFLPase; Act > y > Gal4, UAS GFP; FRT82B tubGal80x UAS-GFP, FRT82B, blue). Oligomycin blocks mitochondrial ATP synthetase, FCCP is mitochondrial ionophore and antimycin A blocks electron transport chain. Time of drug addition is indicated by vertical lines. Hyperplastic discs show higher basal level of glycolysis and lower respiration, accompanied by low glycolytic reserve (increase of ECAR after the block of mitochondrial ATPase with oligomycin) and lower production of ATP in mitochondria (the difference in OCR between basal state and oligomycin-treated conditions), consistent with the induction of glycolytic shift. See Methods for detailed description. Averages of three biological replicate experiments; error bars are standard errors of the mean. Significance is according to one-tailed Student's *t*-test.

Although we demonstrated Notch responsiveness of metabolism-related genes at the transcriptional level, the question remained whether this had any impact on cellular metabolism. To address this point, we measured the concentrations of lactate and fumarate in S2N cells at several time points after 15 min of Notch activation (figure 3*a*). We were able to reproducibly detect a gradual increase in lactate production that coincided with a decrease in fumarate levels. Using isotopically labelled [2-^13^C] glucose tracing and detection by nuclear magnetic resonance (NMR) at the 75 min time point, we observed an increase in extracellular glucose consumption, as well as higher lactate and lower fumarate levels (figure 3*b*).

To test if Notch can mediate metabolic changes in wing discs, we expressed N^icd^ in the flip-out clones that induce hyperplastic growth of the tissue [24] and measured its metabolic parameters on a Seahorse analyser (figure 3*c*). We could clearly see higher levels of extracellular acidification rate (ECAR) in the N^icd^ discs indicating larger amounts of lactate secreted into the medium and hence high levels of glycolysis. In addition, the glycolytic reserve, defined as the increase of ECAR after the block of mitochondrial ATPase with oligomycin, was minimal in N^icd^ discs, suggesting that these cells already run on their maximal glycolytic capacity. On the other hand, their oxygen consumption rate (OCR), indicating the rate of respiration, was lower than in control discs and they produced less mitochondrial ATP (defined as the difference in OCR between basal state and oligomycin-treated conditions). All these metabolic parameters suggest a glycolytic shift in the N^icd^ discs.

Taken together, the metabolic parameters both in cell culture and in wing discs after Notch activation show a metabolic switch towards increased rates of glycolysis and a slower TCA cycle and respiration, thus consistent with the Notch-dependent induction of glycolytic shift.

### 2.4. Notch downregulates tricarboxylic acid cycle via upregulation of the repressor *hairy*

**Figure 4.**
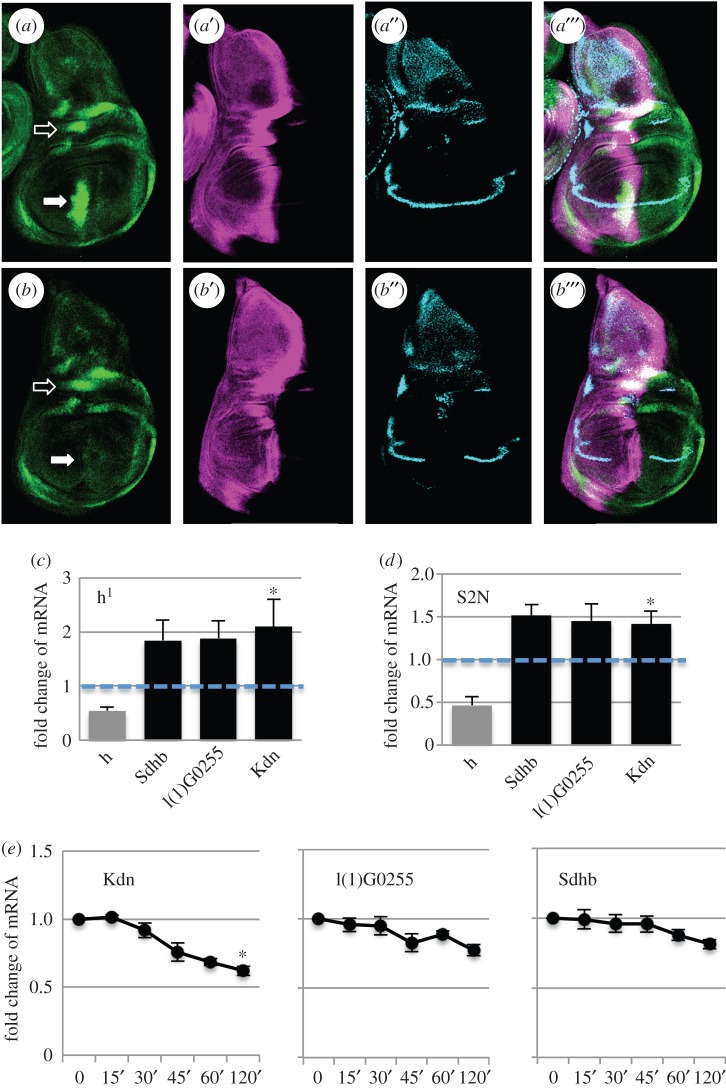
*Hairy* is regulated by Notch and represses TCA cycle genes. (*a*) The expression of *Hairy–GFP FlyFos* construct (green) in control wing discs. *Ci* in magenta, *ct* in blue. Full arrow indicates *hairy* expression in the wing pouch that is Notch-dependent, empty arrow indicates *hairy* expression in the notum that is not regulated by Notch. (*b*) The expression of *Hairy–GFP FlyFos* construct (green) in wing discs with Notch RNAi expressed in the *patched* domain (Ptc-Gal4, tub-Gal80^ts^; UAS-N^RNAi^). *Ci* in magenta, *ct* in blue. The *ptc* domain is slightly wider than the intense band in *Ci* as seen by missing expression of *ct* gene. Full arrow indicates *hairy* expression in the wing pouch that disappeared after N^RNAi^ treatment, empty arrow indicates *hairy* expression in the notum that is not regulated by Notch. (*c*) The levels of mRNA expression of TCA cycle genes with predicted hairy binding sites in *hairy* hypomorphic mutant (h^1^) wing discs in normoxic conditions, relative to the expression in wild-type control *Oregon R* flies. *Hairy* transcript is downregulated to about 50% in these flies (grey column). (*d*) The levels of expression of TCA cycle genes with predicted hairy binding sites after RNAi treatment against *hairy* in S2N cells, relative to the expression in control cells treated with dsRNA against GFP. The efficiency of *hairy* knockdown was 52% (grey column). (*e*) Transcriptional response of TCA cycle genes after 15 min of Notch activation in S2N cells (minutes, as in figure 2). (*c*–*e*) Averages of three biological replicates; error bars shows standard error of the mean. Significance is according to one-tailed Student's *t*-test with three to four independent replicates.

The increased glycolysis after Notch stimulation can, at least partially, be explained by the increased expression of *Glut1* and *Hex-A*, the key genes involved in glucose metabolism. How does, however, Notch stimulation lead to a decreased TCA cycle? The increased expression level of the *Impl3* lactate dehydrogenase gene suggests that at least some pyruvate produced by glycolysis is converted to lactate, rather than being transported to mitochondria and consumed by the TCA cycle. On top of that, there may be another way that Notch downregulates the activity the TCA cycle, through *hairy*. As we presented above, the transcription factor *hairy* is a direct Notch target in S2N cells. To verify if this regulation happens also *in vivo*, we created a *hairy–GFP* FlyFos construct. Its expression in the wing pouch was decreased after Notch RNAi, supporting our previous finding that *hairy* is a Notch target *in vivo* (figure 4*a,b*). On the other hand its expression in the notum was not affected, suggesting its tissue-specific regulation by Notch. However, the FlyFos reporter seems to be more stable than the endogenous protein [27] and thus does not fully recapitulate the endogenous expression pattern of *hairy* (electronic supplementary material, figure S2). This may represent a caveat in the interpretation of the data. *Hairy* has been shown to be upregulated during hypoxic conditions and to act as a metabolic switch by binding to the regulatory regions of several TCA cycle genes and downregulating their expression [25]. To test if it is able to act in similar way even at normoxic conditions, we looked for the expression of TCA cycle genes in *hairy^1^* hypomorphic mutant wing discs of flies kept at normoxic conditions. We saw upregulation of *Sdhb*, *l(1)G0255* and *Kdn* genes which all have predicted *hairy* binding motifs close to their promoters according to Zhou *et al.* [25] (figure 4*c*). We also obtained similar results when we downregulated *hairy* in S2N cells (figure 4*d*). Accordingly, the expression of TCA cycle genes is diminished following Notch activation in S2N cells (figure 4*e*). These results suggest that *hairy* represses, to some extent, TCA cycle genes under normoxic conditions. While *hairy* is our prime candidate for the reduction in TCA cycle genes mediated by Notch signalling in the wing disc, we cannot exclude other mechanisms.

### 2.5. Functional significance of Notch-dependent metabolic changes

Notch signalling controls cell growth and proliferation in several tissues during normal development, including imaginal discs [15] or T-cells [28], and overactivation of the Notch pathway leads to pathophysiological conditions such as wing disc hyperplasia [24] or tumour development [17,29]. Could Notch-dependent upregulation of metabolic genes help to stimulate tissue growth?

**Figure 5.**
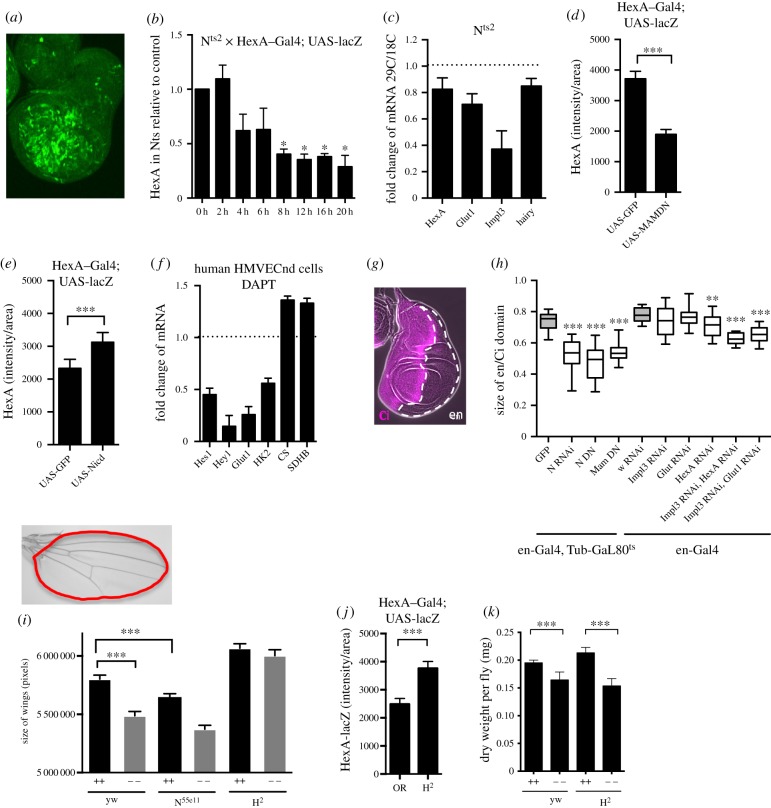
Metabolic genes are Notch targets *in vivo* and in primary human cells and Notch activity can rescue growth in nutrient-deprivation conditions. (*a*) The expression pattern of HexA–Gal4 reporter in the wing discs (crossed to UAS-lacZ). The strength of the signal from HexA–Gal4 reporter was quantified in ImageJ by calculating the integrated density of HexA immunostaining signal from the whole disc (sum of pixel values), after background subtraction, using *Z*-stacks of confocal pictures spanning the whole disc thickness. The integrated density was divided by area of the disc and plotted as ‘HexA intensity per area’. (*b*) The intensity of HexA–Gal4 reporter in wing discs after deactivation of Notch signalling in flies with thermosensitive allele N^ts2^, relative to the expression of HexA–Gal4 reporter in wild-type control Oregon R flies. The *y*-axis represents intensity of HexA reporter per area of the disc, *x-*axis indicates hours after shifting flies from 18°C to the non-permissive temperature of 29°C. Significance is according to one-tailed Student's *t*-test, compared with values at time 0. (*c*) The change of mRNA expression of metabolic genes in wing discs with thermosensitive allele N^ts2^. The ratio of mRNA levels at 29°C against 18°C in N^ts2^ was compared relative to mRNA levels in control wild-type Oregon R flies at the same temperatures. (*d*) The intensity of HexA–Gal4 reporter in wing discs after blocking Notch activation via the expression of dominant negative Mastermind. Control flies express UAS-GFP instead of Mastermind. The *y*-axis represents intensity of HexA reporter per area of the disc. (*e*) The intensity of HexA–Gal4 reporter in wing discs after Notch activation via the expression of Notch intracellular domain (Nicd^MH3^). Control flies express UAS-GFP instead of N^icd^. The *y*-axis represents intensity of HexA reporter per area of the disc. (*f*) The fold changes of mRNA in human microvascular endothelial cells (HMVECnd) after blocking *γ*-secretase with 10 mM DAPT for 6 h, in comparison with cells treated with DMSO. (*g*) Immunostaining of wing disc showing the anterior part (stained with *Ci*, magenta) and posterior part (*Ci* negative, *engrailed*). (*h*) The effect of inhibiting Notch pathway or metabolic genes on the size of *en* domain in wing disc. The thermosensitive form of *Gal80* repressor was used with the *en-Gal4* to drive expression of N^RNAi^, N^DN^, Mam^DN^ and GFP constructs at 29°C for 96 h before dissections of L3 larval wing discs. Two copies of *en-Gal4* driver were used to drive two copies of *UAS-RNAi* of metabolic genes or of RNAi against *white* gene. The ratio between en/Ci domains was plotted. Significance relative to values in control flies (grey). (*i*) The size of adult wings when larvae of indicated genotype were raised on nutrient rich (++) or nutrient poor (−−) food. The N^55e11^ and H^2^ mutants were crossed to *yw* before scoring the heterozygous progeny. (*j*) The intensity of HexA–Gal4 reporter in wild-type control Oregon R and in *Hairless* (H^2^) mutant flies. The *y*-axis represents relative intensity per area. (*k*) Dry weight of *yw* and H^2^ flies raised on nutrient high (++) and nutrient low (−−) diets. Bodies of 20 males with dissected wings were placed per tube, dried on a lyophilizer, and average weight per fly was calculated from three replicates. (*b–e,h–k*) Data from 15 to 30 wing discs or 40 wings; error bars show standard error of the mean or min and max values (*h*). Significance is according to one-tailed (*b*) or two-tailed (*d*,*e*,*h*–*k*) Student's *t*-test.

In order to answer this question, we first tested its obvious presumption that the metabolic genes identified in this study are regulated by Notch. The lack of suitable reporters for *Impl3* or *Glut1* did not allow us to investigate the Notch-dependent regulation of these genes *in vivo* but we could test the regulation of *Hex-A* using a Gal4 enhancer trap line. We used a thermosensitive allele of Notch to reduce Notch signalling and looked for the expression of the *Hex-A* reporter in the wing discs after moving flies from 18°C (with active Notch signalling) to 29°C (with Notch receptor becoming inactive). We saw a gradual decrease of the activity of *Hex-A* reporter in comparison with control (figure 5*b*), corresponding to the decrease of endogenous mRNA for *Hex-A*, *Glut1*, *Impl3* and *hairy* (figure 5*c*). In agreement with this observation, *Hex-A* expression was downregulated when coexpressed with the dominant negative form of Mastermind (figure 5*d*) and it was stimulated as a response to N^icd^ activation (figure 5*e*). As we showed in figure 4*a* and the electronic supplementary material, figure S2D, *hairy* is also a Notch target in the wing disc. Taken together, we showed that metabolic genes are Notch targets *in vivo* in *Drosophila* wing discs.

We also extended our investigations to human microvascular endothelial cells (HMVECnd) that are known to have an active Notch pathway and that exhibit a metabolic profile similar to the Wargurg effect [7]. In this case, we used the *γ*-secretase inhibitor DAPT to inhibit Notch [30] and examined the expression of human *hexokinase 2* and *glucose transporter 1*, as well as of two classic Notch-regulated genes, *Hes1* and *Hey1*, that show sequence similarity to *hairy*. The mRNAs for *Hes1*, *Hey1*, *hexokinse 2* and *glucose transporter 1*, were significantly decreased and mRNA for TCA cycle genes *citrate synthase* (*CS*, human orthologue of *Drosophila Kdn*) and *succinate dehydrogenase* (*SDHB*) increased under these conditions (figure 5*f*). Such changes in expression could result in lower glycolysis and increased TCA cycle, hence reversal of the Warburg effect phenotype. These observations fit with the hypothesis that Notch is needed for the maintenance of the Warburg effect in these cells, in full agreement with our *Drosophila*-based model.

As the growth of the wing disc is dependent on Notch signalling [15] and we showed that Notch controls the expression of metabolic genes in this tissues (figure 5*b–e*), we asked whether downregulation of metabolic genes would lead to smaller growth of the wing disc. Simultaneous downregulation of *Hex-A* and *Impl3* or *Impl3* and *Glut1* using *en*-Gal4 driver caused significant reduction of the posterior compartment (figure 5*h*), similar to the phenotype observed when downregulating Notch signalling [31]. While these *in vivo* experiments point towards the model where Notch-dependent growth is supported by upregulation of metabolic genes, they do not unambiguously distinguish between the direct and indirect effects of metabolic gene regulation by Notch. However, our cell-line-based data point towards this idea.

A similar effect was observed in adult wings. Here, instead of downregulating metabolic genes by RNAi, we slowed metabolism by keeping flies on low-nutrient food. Wings of *N^55e11^* heterozygous mutants were smaller than wild-type, similar to wings of wild-type flies kept on low-nutrient food. On the other hand, upregulating the Notch pathway in mutants for the Notch repressor protein *Hairless* leads to larger wings. Surprisingly, *hairless* mutant flies kept the size of their wings unchanged on low-nutrient food (figure 5*i*). This was not because H^2^ larvae would spend a longer time as L3 and have more time to develop in to overall bigger/heavier flies through a systemic regulation of body growth (electronic supplementary material, figure S3B and 5K). Instead, only the Notch-dependent growth of the wings was affected without having an effect on the weight of the rest of the body that is Notch independent. Our model would predict that *hairless* mutant wing discs upregulate metabolic genes and this level of upregulation is sufficient to maintain proper disc development under conditions of low-nutrient availability (increased expression of *Glut1* promotes sufficient uptake of glucose even in conditions of low-nutrient availability and increased expression of *Hex-A* and *Impl3* promotes glucose utilization). Indeed, the level of *Hex-A* reporter was upregulated in the *Hairless* mutant (figure 5*j*) and *Glut1*, *Hex-A*, *Impl3* and *hairy* were upregulated after *Hairless* RNAi in S2N cells (electronic supplementary material, figure S3A). Taken together, our data are in agreement with a model where Notch-dependent regulation of metabolic genes, direct or indirect, is able to stimulate growth of the wing disc, and that in this way Notch is able to buffer fluctuation in nutrient supply to support proper wing development.

**Figure 6.**
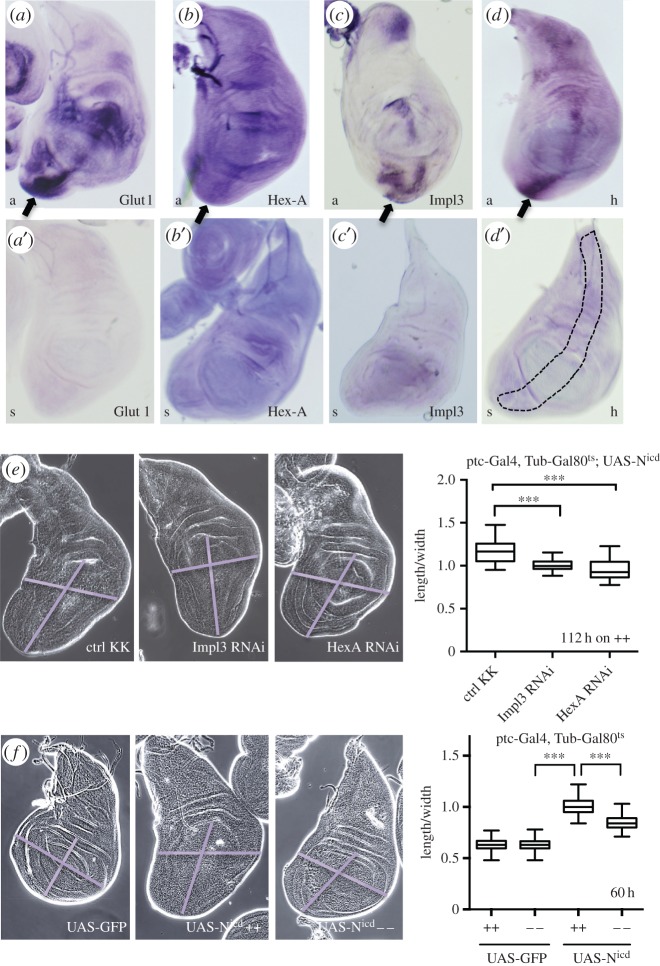
Upregulation of metabolic genes is important during Notch-induced hyperplasia. (*a*–*d*) *In situ* hybridization of Notch-mediated hyperplastic wing disc (using *ptc* driven *Su(H)-VP16*) with specific antisense probes (‘a’, in *a–d*) or negative control sense probes (‘s’ in *a*′–*d*′).The *ptc* domain is schematically indicated in figure *d*′. (*e*) Larvae with N^icd^-induced hyperplasia in the wing disc (Ptc-Gal4, Tub-Gal80^ts^; UAS-Nicd^MH3^) crossed to control KK line, Hex-A RNAi or to Impl3 RNAi, kept at 29°C for 112 h before dissection on high nutrition food (++). The size of wing pouch was quantified by measuring the ratio between the length and width of the pouch. (*f*) Larvae with N^icd^-induced hyperplasia in the wing discs (Ptc-Gal4, Tub-Gal80^ts^ crossed to UAS-Nicd^MH3^ or control UAS-GFP) were raised on nutrient rich (++) or nutrient poor (−−) food and the size of wing pouch was quantified by measuring the ratio between the length and width of the pouch. (*e*,*f*) Data from 23 to 91 wing discs; error bars show min and max values. Significance is according to two-tailed Student's *t*-test.

Finally, we asked whether we could see upregulation of metabolic genes during Notch-induced hyperplasia and whether we could suppress this phenotype by downregulating metabolism. We used the *Su(H)-VP16* construct (thus making Su(H) a constitutive activator) to overactivate the Notch pathway in the *patched* domain of the wing disc epithelium that is known to induce cell overproliferation, especially in the ventral top region of the disc [24]. We analysed the transcriptional response of metabolic genes in this tissue using *in situ* hybridizations. Although this technique lacked the sensitivity to unambiguously describe the endogenous expression patterns of our candidate genes across the wing disc, the upregulation of *Glut1, Hex-A, Impl3* and *hairy* in the *patched* domain was clearly evident and especially profound in the most proliferating ventral top region of the disc (figure 6*a*–*d*). Importantly, we were able to partially rescue Notch-dependent overproliferation by downregulation of *Hex-A* or *Impl3* expression (figure 6*e*) or by keeping larvae on low-nutrient food (figure 6*f*).

**Figure 7.**
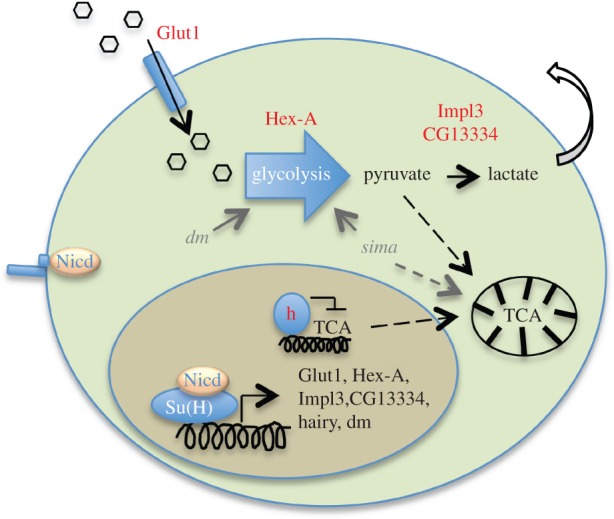
Model of Notch response leading to changes in cellular metabolic profile. Schematic model of the Notch response leading to increased glycolysis and decreased TCA cycle via the direct transcriptional regulation of metabolic genes. Collaboration with *myc (dm),* that is also a Notch target, or with *Hif-1* (*sima*) in the case of hypoxic conditions may help the metabolic transition.

Based on our results, we suggest a model where Notch-dependent upregulation of metabolic genes contributes, among other mechanisms, to the stimulation of cell growth in the wing disc during normal development as well as during Notch-induced hyperplasia (figure 7).

## 3. Discussion

In this study, we identified several genes involved in metabolism to be transcriptionally regulated by Notch, both in cell lines and *in vivo*. This way Notch activation helps in shifting the cellular metabolic status towards increased rates of glycolysis and lower TCA cycle in a manner reminiscent of the Warburg effect. This metabolic transition may be relevant in various contexts where Notch activation stimulates growth, proliferation or cell survival.

Glycolytic shift was previously observed in cancer cells with hyperactivated Notch signalling, where the induction of metabolic genes relies on the PI3K/Akt pathway [19]. Our results point to a more general model where regulation of metabolic genes by Notch is not restricted to cells with overactivated Notch receptor but happens also in cells with endogenous levels of Notch signalling. Therefore, regulation of metabolism by Notch may play an important physiological role in various situations during development or adult homoeostasis. Indeed, recent evidence shows that Notch-mediated survival of memory CD4+ T cells is dependent on the regulation of glucose uptake through the expression of Glut1 [32]. Glycolytic phenotype is essential for the quiescence and self-renewal of adult stem cells [33] as well as for the rapidly proliferating embryonic stem cells [8,34], and Notch has been shown to be critical in both of these processes [35] although the direct regulation of metabolism by Notch has not yet been investigated in this context. Cells during immune response also switch to glycolytic metabolism [36], and Notch has been shown to govern differentiation of distinct haematopoietic cell types [37] but again, whether Notch can directly promote metabolic shift in these cells has not been investigated.

The Warburg effect is usually also associated with increased activity of the pentose phosphate pathway (PPP). We attempted to measure the fraction of glucose that goes through PPP using [2-^13^C] glucose [38], but the spectra for PPP derived [3-^13^C] lactate overlapped with alanine and we were unable to accurately determine the PPP flux. Although the low activity of the TCA cycle also suggests a metabolic shift towards the Warburg effect, we cannot exclude that Notch activation stimulates glycolysis without an increase in PPP.

We see Notch-dependent downregulation of the TCA cycle (figures 3*a*–*c*, 4*c*–*e* and 5*f*) and we propose *hairy* as the candidate transcription factor mediating this repression. We see upregulation of *hairy* in Notch hyperplastic discs (figure 6*d*); however, the hairy-FlyFos-GFP reporter we used to determine *hairy* regulation by Notch in normal wing disc development does not fully reproduce the endogenous *hairy* expression pattern in the wing pouch using *hairy* antibody. Thus, the FlyFos reporter may not truly reflect endogenous regulation of *hairy* that is known to be unstable on the level of protein as well as mRNA [27]. While *hairy* is our prime candidate to explain how Notch downregulates TCA, other mechanisms are also possible. At the same time, in certain context, the upregulation of glycolytic genes may not coincide with upregulation of *hairy* and, therefore, the Notch-dependent regulation of glycolysis may happen independently of the regulation of the TCA cycle.

It is remarkable that the relatively low and transient changes in the mRNA expression of the Notch-regulated metabolic genes identified in our study are associated with such profound changes in the cellular metabolic profile. However, changes of similarly small magnitude have been reported to promote the Warburg effect in cancer cells. For example, in trastuzumab-resistant human breast carcinoma cells, a 2.5-fold induction of Glut1 and 0.5-fold induction of Ldha (lactate dehydrogenase) correlated to increased glucose uptake and increased production of lactate [39]. Likewise, a 2.5-fold upregulation of Glut1 and 0.5-fold increase in hexokinase 2 caused by downregulation of Hsulf-1 in ovarian cancer cells lead to glycolytic phenotype [40]. In human bladder cancer cells, the expression of a long non-coding RNA UCA1 promoted glycolysis via a 0.5-fold increase in hexokinase 2 expression [41]. Our data are therefore fully consistent with these studies in human cells, which lend further support for the functional significance of our results. We can speculate that the transcriptional changes after a relatively short pulse of Notch activation (15 min) lead to increased protein levels that are then stable over a longer time period and thus cause the metabolic changes we observe. How long this metabolic shift lasts and whether sustained or repeated Notch activation would be necessary to maintain it remains an open question. However, our data show that even 75 min after the initial Notch pulse, lactate production is still increasing despite the fact that the mRNA for *Hex-A* and *Impl3* have long returned to their original levels (figure 2).

We show that metabolic genes are Notch targets in the wing discs and decreased Notch signalling has similar phenotypic consequences on growth as decreased expression of metabolic genes. Moreover, we rescued the Notch-driven overgrowth of the wing disc by downregulation of glycolytic genes. These observations are consistent with the model where Notch is able to stimulate cell growth during *Drosophila* wing disc development by the upregulation of metabolic genes. Our *in vivo* data do not unambiguously distinguish between the direct or indirect regulation of metabolic genes by Notch, but our cell-line-based data point towards direct effects. Notch has been shown to regulate the growth of wing discs by several other mechanisms, mainly through the regulation of *Wg* signalling, the transcription factors *vg* and *myc* and cell cycle regulators [24,42]. Although we did not directly measure the metabolite levels in wing discs, the transcriptional regulation of metabolic genes suggests that Notch helps to establish or maintain glycolytic shift in this tissue. Metabolic regulations may also be part of the Notch-induced proliferation in other contexts.

The regulation of metabolic genes to stimulate tissue growth is certainly not a privilege of Notch. The growth of wing discs is primarily driven by Notch, but in other contexts metabolic genes might be induced by other signalling pathways via different enhancers in their regulatory regions or indirectly through the introduction of transcription factors such as myc. Specific chromatin states could determine whether metabolic genes will be responsive to Notch or to a different signal or transcription factor, in a tissue- and context-specific manner.

The metabolic genes mentioned in our study are not the only ones that Notch may use to mediate changes in cellular metabolism. The transcription factor *myc* is a direct Notch target but also a master regulator of the cell cycle and growth, stimulating the transcription of ribosomal genes as well as enzymes of glycolysis, glutaminolysis and lipid synthesis [17,43]. *Hif-1* is the key regulator of metabolism under hypoxic conditions, such as in cancer, and its targets also involve genes of glycolysis and glutaminolysis. Mouse NICD1 can physically interact with HIF-1 and potentiate its recruitment to its target promoters [44]. It is therefore possible that the Notch pathway uses several mechanisms to promote glycolytic shift, one of which is the direct transcriptional upregulation of metabolic genes that we describe.

It is apparent form our results that low-nutrient availability cannot rescue the mild wing overgrowth of H^2^ mutants but it can rescue *ptc* *>* *N^icd^*-driven wing disc overgrowth. These results appear contradictory, however they represent very different conditions. In the former, the reduction in *Hairless* results in very mild increase in Notch activity that is likely to be within a range where homoeostatic buffering can occur. For example, a negative feedback loop limits the extent of Notch pathway activation and restricts expression of metabolic enzymes to a certain threshold, leading to mild disc overgrowth. In the *ptc* *>* *N^icd^* discs, there is sustained high level of Notch activity that is no longer subject to the normal feedback regulation (as it is controlled by exogenous Gal4 system). Many genes are upregulated under these conditions [24], which are likely to circumvent the normal feedback regulation allowing maximal metabolic rates and massive overproliferation.

## 4. Conclusion

Our results indicate that a short pulse of Notch signalling is sufficient to induce glycolytic shift by direct transcriptional activation of genes involved in glycolysis and regulation of the TCA cycle. We propose that direct regulation of metabolic genes is one of the mechanisms by which Notch promotes tissue-specific growth during development as well as during hyperplasia. Notch-mediated metabolic reprogramming may therefore be a common tool that helps Notch to exert its effects in target tissues.

## 5. Methods

### 5.1. Luciferase assay

Genomic regions covering Su(H) ChIP peaks were cloned into pGL3 vector containing a minimal promoter (pGL3-basic vector from Promega where we cloned 110 bp of hsp70 promoter into *Bgl*II, *Hind*III sites). S2 cells were transfected with FuGene6 (Roche) together with a copper-inducible N^icd^ (pMT-N^icd^) and a normalizing Renilla construct (pRL-TK vector from Promega that provides constitutive expression of Renilla luciferase). The empty pMT vector was used in controls to substitute for N^icd^. Cells were harvested 24 h after the addition of 600 µM CuSO_4_ and measured by the Dual luciferase reporter assay system (Promega). The restriction sites used for cloning as well as the sequence of the cloned genomic regions including the positions of the Su(H) sites and their mutated versions can be found in the electronic supplementary material. The positive (M3) and negative (NME) constructs are described in Li *et al.* [45].

### 5.2. Notch activation in S2N and DmD8 cells

S2N cells are a stable Notch-expressing S2 cell line containing a Cu^2+^-inducible (metallothionein promoter) pMT–Notch construct [46] and grown in Schneider medium (Life Technologies) supplemented with 10% foetal bovine serum (Sigma) and penicillin/streptomycin (Sigma), under permanent selection with 10 µM methotrexate (Sigma). Expression of full-length Notch was induced overnight by 600 µM CuSO_4_ (Sigma) in cell culture medium. DmD8 cells were obtained from the Drosophila Genomics Resource Center and grown in the same medium as S2N cells without methotrexate and supplemented with 10 µg ml^−1^ insulin (Sigma). The Notch pathway was activated by 2 mM EDTA in PBS as in Krejci & Bray [26] for 15 min then replaced by fresh medium, and cells were harvested at appropriate time points. Cells were treated with 5 µM *γ*-secretase inhibitor compound E (Abcam) for 16 h before activation and during the time course and with 100 µM cycloheximide (Sigma) or 50 µM LY294002 (Abcam) for 1 h before activation and during the time course. Appropriate vehicle was used in controls (DMSO or ethanol only).

### 5.3. RNAi treatment of S2N cells

Double-stranded RNA for *hairy* was produced using PCR templates with T7 promoters attached to their ends (taatacgactcactataggg TGCTACAGCACCTGAGCAAC and taatacgactcactataggg ATGTGTGCGAGTTGGATGAG) and transcribed by RiboMax system (Promega). Cells were seeded into six-well plates, medium was replaced by 300 µl of Optimem containing 20 µg of dsRNA, and cells were incubated for 30 min followed by addition of fresh Schneider medium. Forty-eight hours later cells were treated with dsRNA again following the same protocol and harvested after the next 48 h.

### 5.4. Human microvascular endothelial cells from neonatal dermis cells

Human microvascular endothelial cells from neonatal dermis cells (HMVECnd) were purchased from ThermoFisher Scientific and grown at 37°C in a humidified atmosphere containing 5% CO_2_, in Medium 131 containing penicillin/streptomycin and Microvascular growth supplement (all from ThermoFisher). DAPT (*N*-[*N*-(3,5-difluorophenacetyl)-l-alanyl]-*S*-phenylglycine *t*-butyl ester) was purchased from Sigma.

### 5.5. mRNA level measurement

RNA from 10 cm dishes of cultured cells was extracted in 1 ml of Trizol (Sigma), RNA from 60 wing discs using 0.5 ml Trizol. RNA was treated with DNAse (DNA Free reagent from Ambion), reverse transcribed with MMLV reverse transcriptase (Sigma), and specific mRNAs were quantified by real time PCR using GoTaq qPCR master mix (Eastport Scientific) run on a BioRad CFX96 machine. Primers were designed to not span introns, and a calibration curve from serially diluted genomic DNA was used in every run to accurately quantify the cDNA. Values were normalized to the *CG11306* gene (see electronic supplementary material, figure S4).

### 5.6. Measurement of extracellular acidification rate and oxygen consumption rate

The Seahorse metabolic flux analyser (model XF^e^24) was used to measure the metabolic parameters in wing discs of abx UbxFLPase; Act > y > Gal4, UAS GFP; FRT82B tubGal80x UAS-Nicd; FRT82B or control discs crossed to UAS-GFP; FRT82B at 25°C. Discs were dissected and measured in bicarbonate-free Schneider medium containing 11 mM glucose and 12 mM glutamine (Sigma S9895) using islet plates, and data were normalized to protein content using the BCA assay kit (Sigma).

During glycolysis, lactate is secreted into the extracellular space leading to the acidification of the medium. The rate of glycolysis can then be assessed by measuring the ECAR (changes of pH of the medium per time interval). As oxygen serves as final electron acceptor at the mitochondrial respiratory chain, the rate of respiration can be measured by monitoring the oxygen consumption rate (OCR, changes of O_2_ concentration per time interval).

We measured ECAR and OCR in the wing discs for an initial period of 36 min, where basal state of ECAR and OCR was determined at seven time points (each consisting of 1 min of sample mixing, 2 min of waiting and 3 min of measurement). Then, 5 µM oligomycin was injected in to the cells to block the mitochondrial ATPase that in control cells should lead to lower respiration (OCR) compensated by higher glycolysis (ECR). After four cycles of ECAR and OCR determination, the H^+^ ionophor FCCP (2 µM, carbonyl cyanide 4-(trifluoromethoxy)phenylhydrazone) was injected into the cells to depolarize the mitochondrial H^+^ gradient leading to higher oxygen consumption in control cells. After four cycles of OCR determination, 5 µM antimycin A was injected to block completely the electron transport chain and formation of membrane H^+^ gradient (inhibiting the reduction of ubiquinone by binding to the Qi site of cytochrome *c* reductase) that was reflected in a sharp decrease in OCR below the basal level. Oligomycin, FCCP and antimycin A were purchased from Sigma. ECAR and OCR values are normalized to protein levels in individual cells using the BCA method (Sigma kit, BCA1), after lysis of well content in RIPA buffer.

### 5.7. *Drosophila* strains and induction schemes

C-terminal fusion of *hairy* with eGFP was created in the 43 kb FlyFos031638 fosmid clone following the protocol described in Ejsmont *et al.* [47] using S0062-R6 K-2xTY1-eGFP-FNF-3xFLAG as the tagging vector (gift from M. Sarov). The fosmid was injected into embryos carrying the attP40 site for stable transgene integration. Flies carrying this construct were crossed to Ptc-Gal4, tub-Gal80^ts^; UAS-N^RNAi^ [48] and kept at 29°C for 72 h to downregulate Notch signalling in the *ptc* domain of the wing disc. The Gal80^ts^ is a thermosensitive allele of Gal80 repressor that blocks the function of Gal4 until it is shifted to 29°C where Gal80 is not functional. h^1^ flies (BL513) were raised at 25°C. To generate N^icd^ flip-out clones leading to wing disc hyperplasia, we crossed abxUbxFLPase; Act > y > Gal4, UAS GFP; FRT82B tubGal80x UAS-Nicd^MH3^; FRT82B and kept the cross at 25°C until L3 larvae emerged [24]. Notch-induced overgrowth in figure 6 was achieved by crossing Ptc-Gal4, Tub-Gal80^ts^ with UAS-Nicd^MH3^ [24], keeping the progeny at 18°C until the appearance of early L2 larvae and then moved to 29°C for 60 h (E) or 112 h (F) before the dissections of L3 larvae. Flies with the thermosensitive allele of Notch N^ts2^ (BL3075) were kept at 18°C to allow normal N^ts2^ function and at indicated times before dissections larvae were shifted to 29°C to block Notch activity. Crosses using UAS-MAM^DN^ (BL26672), UAS-N^RNAi^ (BL7078) and UAS-N^DN^ [50] flies in figure 5 were kept at 18°C and induced at 29°C for 20 h (figure 5*e*) or 96 h (figure 5*h*) before the dissections of L3 larvae. UAS-N^icd^ flies were kept at 18°C and induced at 29°C for 72 h before L3 dissections. Flies with double en-gal4 driver and double RNAi transgenes were created by recombining both the elements on the second chromosome and used either as homozygous stocks or crossed to another line with en-gal4 and RNAi on the second chromosome. Fly progeny was kept at 25°C for 40 h and then at 29°C for 120 h before the dissection of wing discs from L3 larvae. *Ci* domain was stained with *Ci* antibody and the size of *en* domain was determined as the part of the wing disc lacking signal of *Ci* staining. The following RNAi lines were used: Impl3 (VDRC 102330), Hex-A (VDRC 100831), Glut1 (VDRC 108683), control KK line (VDRC60100, y,w[1118];P{attP,y[+],w[3′]}), white (BL35573). All larvae used for immunostainings were carefully staged, only early L3 larvae that came out of food within a 2 h window were used for dissections. Additional flies used in the paper were HexA–Gal4 enhancer trap (DGRC 105136), H^2^ (BL517 over TM6 balancer), N^55e11^ (BL28813). The strength of the signal from the HexA–Gal4 reporter was quantified in ImageJ by calculating the integrated density of HexA immunostaining from the whole wing disc (sum of pixel values) divided by area of the wing discs. *Z*-stacks of confocal pictures spanning the whole disc thickness were used. Background was substrated and value plotted as ‘HexA intensity per area’. Size of *en* domain was determined as the part of the disc lacking signal of Ci staining.

### 5.8. Antibodies

*LacZ* (40–1A), *Ci* (2A1) and *cut* (2B10) antibodies were obtained from the Developmental Studies Hybridoma Bank. *GFP* antibody (ab290) and *hairy* antibody (ab20165) were from Abcam.

### 5.9. *In situ* hybridizations

Digoxygenine-labelled RNA probes were produced using a PCR template with T7 promoter attached to the 5′-end and *in situ* hybridization was performed as described in the electronic supplementary material. *Su(H)-VP16* flies [50] were crossed to *ptc-Gal4, Tub-Gal80^ts^*, moved to 29°C in second instar to induce *Gal4* expression and dissected 2–3 days later.

### 5.10. Measurement of metabolites by nuclear magnetic resonance

S2N cells were seeded in Schneider medium and Notch expression induced overnight by 600 µM CuSO_4_ (no CuSO_4_ in control cells). Cells were activated by EDTA for 15 min, then either the 15 min time point was harvested or fresh medium was added and cells harvested at indicated times (minutes). Cold methanol extraction and metabolite quantification was performed as in Sellick *et al.* [51]. All spectra were acquired at 25°C and processed using TopSpin v. 3.2 (Bruker, USA). Signals intensities were normalized to total protein concentration. Additional details of the method can be viewed in the electronic supplementary material. For experiments with isotopically labelled [^2-13^C] glucose in figure 3*b*, cells were activated for 15 min by EDTA after which medium with [^2-13^C] glucose was added and cells harvested at the 75 min time point, followed by acetonitrile extraction and metabolite measurement by NMR as in Delgado *et al.* [38].

### 5.11. High- and low-nutrient food

The high-nutrient food contained 160 g yeast and 160 g glucose per litre, the low-nutrient food contained 20 g of yeast and 20 g glucose per litre. L1 larvae from staged cages were transferred to the appropriate food and let develop at 25°C. To assess the body weight of flies on different food, we dissected wings from 20 male flies, placed their bodies into a preweighted Eppendorf tube and lyophilized. Averages of triplicate measurements were plotted.

### 5.12. Statistical analysis

Statistical tests were performed as described in the Figure legends. *p ≤ 0.05, **p ≤ 0.01, ***p ≤ 0.001.

## Supplementary Material

Supplementary Material
