# Supplementary material for: Notch stimulates growth by direct regulation of genes involved in the control of glycolysis and the tricarboxylic acid cycle
Source: Open Biol. 2016 Feb 17;6(2):150155. doi: 10.1098/rsob.150155 (PMC4772804; doi:10.1098/rsob.150155)

Supplementary Material

Supplementary Figure 1:

***CG13334* has predicted Su(H) sites and responds to Notch in luciferase assay.**

A. Genomic region of *CG13334* showing Su(H) ChIP peaks. Legend as in Fig.1A, black peaks are from *DmD8* cells.
B. Luciferase assay in S2 cells using genomic regions indicated in panel A. P-values according to Krustall-Wallis test comparing groups against NME.
C. Comparison of Notch mRNA levels in uninduced *S2N* cells (no Cu), induced *S2N* cells (Cu) and *DmD8* cells**.**

Supplementary Figure 2:

The expression pattern of hairy

A. The expression pattern in wild type control Oregon R wing discs using anti *hairy* antibody (Abcam 20165)
B. The expression pattern of *hairy-FlyFos* construct tagged with GFP stained with GFP antibody.
C. The expression pattern of *Hairy-Gal4* (BL30876 y^1 w^*; PBac{h-EGFP.S}VK00037 x UAS-GFP) using GFP antibody.

Hairy is expressed in the pouch as well as in the notum of the wing disc. While the relative intensities of the two bands in the pouch (arrows) differ depending on the reporter used (possibly due to a different timing and perdurance of the constructs) the overall expression pattern is preserved, although not fully recapitulated.

Supplementary Figure 3:

Additional data related to Hairless.

A. S2N cells were treated with dsRNA against *Hairless* and the expression of mRNA for metabolic genes were quantified relative to their expression in control cells treated with dsRNA against GFP (mock treated, first column). Normalized to the housekeeping gene CG11306.

B. Time (hours) of pupation of H^2/+ and wildtype control (yw) flies. Thirty L1 staged larvae of indicated genotype were placed per one vial on nutrient rich (++) or nutrient poor (--) diets and pupated adults were counted at indicated time points after egg laying (AEL). 90 larvae were counted per genotype.

Supplementary Figure 4:

The comparison between mRNA quantification data using rp49 or CG11306 as normalizing genes

A. Single experiment from cycloheximide treatment of S2N cells as in Fig.2B, normalized to rp49. Control cells in black, cycloheximide treated cell in green. Normalization to rp49 gives very similar trends as normalization to CG11306.

B. Single experiment from Fig.4D. Normalization to rp49 or CG11306 gives nearly identical results.

<u>Files that will be part of electronic supplementary material (ESM):</u>

ESM1: Sequences of enhancers tested in luciferase assay

Sites for cloning into pGL3 with minimal promoter are in brackets, mutated Su(H) binding sites are marked in red, followed by their mutated versions.

Glut1 *(Kpn1, Bgl2)*

gaagacgacgacatgactgccttttattgctcctacgcctctgcctagcccctccgttcccatttttcccacgcccct
ccttttgttcagctgtcgtggccgttgaacaaatgaacaacataaataattgcagaggcacacacactgacac
acacacacacacgcgttctgaggaaactcgcacgcacactgacaaagagtgggaaaatcgcaggactcccaa
ggaacgtttgcagccttcgaattgagattaatgagcagcaaaagtgattcaaagtcagcatcctcg

ttcccac → TAACAAC

Hex-A (1) *(Kpn1, Bgl2)*

tgtgctacaagcgaaagcagacatcgcaagtataacagttgaaggtgacactggagaccctggcgataggac
acgtataacagttctgtaatcccaacattggctgacgaaatcatgggaatgcaaggcgtggttgggcgggttaa
gccacttctcccaaaaagttataaaaacccatctggaaccagttgatttttgtatttatctggttctgttgacatgg
tgattttacttcaaagaacccgtttatgaaattcgaaacctacaatgtaaatggaaattccctgagtcaatttttcc
aatatgcaactcctttgga

atgggaa →ATTGTTA

ttctccc →TAATACC

Hex-A (2) *(Kpn1, Bgl2)*

cagcaccgaatggaaattgcacatgaaatctagctgcaaaaatatgaaacaacaaaattccattgaatggaga
gccagaaagagcgaagtagaggggaagagagtgagagaaggagggagcggaacttcaaactgggcggcac
gtacacacacaaaagagggcagggcggggagacgagccccacggacactgaacaaaaatacatacaaatgc
aaagatatatgtatttgaggatgttgacgcgtcgctagaacttgagaattccaagaagcggtaaaaacgggtga
gacgatgatggtaggggagttggggaggggaaggtagctgtcaacagctacgcaaacttgctctcccatttttc
tgggtaaaccttgctctttctcatctaaaacggcattgttatactaacaccgatgccgagggagagcgagatcgc
tgagagtgggaaagcgggtggtggggcccggcgccgaacagggggttcaaagaacgaagccaa

gtgggaa →GTTGTTA

Hex-A (3) *(Kpn1, Bgl2)*

gcgacgcataagggtttccgcataaacacgcgtttgactcattctcattcatagccactctctcgccccctctctca
cgcgcaccctatcaatatgccatttgtggacccctcagcgtcgcacatatacacgccatatctccaaccatgccg

ImpL3 (Kpn1, Bgl2)

Tcagtttcgtttggggagagctaaactcaagaccaactgaagttcacttcagttttcagctcccgagcagcagca
gcagcagcaactttcgagatatacgagcatttgtcacataaaactgcaacgcctttcccattgtcagctcgccac
gaagacttggtcgctgtttgcttttgcttttggccataaaatatatggccccgtatcgcaaaaagctgcgagtgtt
gaaatttaagttttaatttaagcgatatgaaaaaaaaagggcaagacggcaaacggcggtctgcagtcgtcga
atcgtgcacgaaatgaagccattttcatatttatgaattttgccccttatcgaaaaacgcttcaattggccatcgac
ttggggccggcaaattaaatatgggcagtcataaacgatactgacaggtcggtgggatctgtgggaaaagggg
caagattccggggaacttggagatccagaatccagagcgtaacatcctcgccgacttagctgagttattaaggc
atctttatagccaatctcaatgaggtgtgaagtgcaatcgttgttggcctttttattaatacgcgaaaatatatttta
tttgttgttccgtctgcgatcgactgcgatattaagc

gtgggaa → GTAGAAA

hairy (Mlu1, Bgl2)

agcaacaacaccaacaccaccgcgaccatcaccaacagcacagccagaaacacagcctcttgtgaatccctca
gttagcagagcccagcagagtcaagccaaaccgatcgctgatcgaccgaccgaccgacgatcaccaatgggg
gtttcgcagtgtgatttccaaaaaggaagaaatgcccattcccgcgagccacgggggcgtatgagtaacgcggt
g

ttcccgc → TAACAGC

CG13334 (1) (Kpn1, Bgl2)

ctgctccatttgctgttgacctattgaaatgcgacagtttcacatgtgttttcgggattgcgtgggatgatcgtgga
gcacgtgtgtgttgacatactgaccgcagagagtgtacttgagcaagggacttccccgtgaatctgcccgcctgc
tcaagtgctacgtaatttgcatggtctggatttttcaaattacatgacattttcccacactgattagcccgcacat
gccattctctattttcgatgtgcatccgagtgcgaccttcgaacaggcaaacagccagtgcagcctgcatccgat
gcacatccattgcatccagtgcaacgaagtgcaacgccatcatttttcccacagccgagggctctgggttatatt
taacaaatgcatgagagatttgggtgcgctacgttttcgttttcgtttcgga

CG13334 (2) (Mlu1, Bgl2)

ccctggatacagacgattgcgctccactgatttttcccatcatcgagcgatgctccctgcctttgggcactttcttc
atctcctggctggcctcctgcaatttgctgtgaacctgttcgaaaattatgcatcgatgcgcatcagtggcaatca

tggatggcaacataataatgggatgctgctgatgagtttgctcataaactgatgagggtacctggctgtctacct
agctacctgtacctgtgattcatttcgattgcctagctgctgattctgttcacctgttcgtttgctacactttatgcat
gcttcggcagttttctaattatgacttatgatctctgggattagacaaacaatggatcgtggcacgtggttcccaa
gatgctttcgcttgtgcctgaccttttcccacgatctgtttgcggttgagcgctcgaggcccatgatcaccgcaaat
cccctgcagacaaaacagccaaagg
ttcccac → TAACAAC

CG13334 (3) (Mlu1, Bgl2)
tggacgcaaccatcatattcttctgcgctcctgatccttttttccaatacctcgattcggagttctcacgcgagcaca
tgccaaagcgaaaaaaagggacagggaaacaattaacaactaatttaggcctctgcatataattttatggaga
gtggtgcggagagtcgcagtcccacagggagatgcctatctgcaggagctgtgggaaaaaatacgttacattc
ctttcctttgctttccttctctctctgcctgtcgttgcggcatttattcccacgcgatcctcgactctcgtttttgttggt
tttcggggtttcgctttcgtggagta

ESM2: Primers for in situ hybridizations

Impl3 sense probe
T7 ImpL3 s GAATTAATACGACTCACTATAGGGAGA
GTGTGCCTCATCGATGTCTG
ImpL3 probe a CCCAGGAGGTGTATCCCTTT

Impl3 antisense probe
T7 ImpL3 a GAATTAATACGACTCACTATAGGGAGA
CCCAGGAGGTGTATCCCTTT
ImpL3 probe s GTGTGCCTCATCGATGTCTG

Hex-A sense probe
T7 Hex-A IS s TAATACGACTCACTATAGGG TGTGTACAAGGAGCGTTTGC
Hex-A IS a GGCTCGTCAGCTTCAATT

Hex-A antisense probe
T7 Hex-A IS a TAATACGACTCACTATAGGG GGCTCGTCAGCTTCAATT

Hex-A IS s TGTGTACAAGGAGCGTTTGC

Glut1 sense probe
T7 Glut1 IS s TAATACGACTCACTATAGGG GGAGATAGCGCCACTGAA
Glut1 IS a ATTAGCGGAATGGACACGAG

Glut1 antisense probe
T7 Glut1 IS a TAATACGACTCACTATAGGG ATTAGCGGAATGGACACGAG
Glut1 IS s GGAGATAGCGCCACTGAA

hairy sense probe
T7 hairy IS s TAATACGACTCACTATAGGG TGCTACAGCACCTGAGCAAC
hairy IS a ATGTGTGCGAGTTGGATGAG

hairy antisense probe
T7 hairy IS a TAATACGACTCACTATAGGG ATGTGTGCGAGTTGGATGAG
hairy IS s TGCTACAGCACCTGAGCAAC

ESM3: Supplementary description of methods

***In situ* hybridizations**

Sense and antisense digoxygenine labeled RNA probes were produced using specific PCR products with T7 promoter sequences at their 5'ends using DIG RNA labeling mix (Roche). Primer sequences can be found in Supplement 2. Proximal halves of larvae containing brains, imaginal discs and other tissues were dissected in PBS and fixed by 4% formaldehyde for 30 minutes, washed with 50% hybridization solution (HS, 50% Formamide, 5X SSC buffer, 100μg/ml DNA salmon sperm, 50μg/ml Heparine, 0.1% Tween20) in PBT-Tween 0,1% for 5 minutes and stored in -20°C in HS. Samples were then incubated with equal amounts of antisense or sense probe in HS at 60°C over-night, washed with HS and HYBE solutions (50% Formamide, 50% 5X SSC buffer), signal detected by anti-DIG antibody (Roche) and wing discs

dissected and mounted in glycerol.

Metabolite measurement by NMR

Cells were seeded in Schneider media and Notch induced by 600µM $CuSO_4$ overnight. In the morning cells were washed twice with PBS and collected into cold methanol (-20ºC). The samples were then spun (14,000g/5min) – both supernatant and pellet were collected. The pellets were used to establish the protein concentrations using standard Bradford protein essay. The supernatants used for preparation of NMR samples were processed as follows: solvent was removed from the supernatants by controlled evaporation under lowered pressure and room temperature. Dry pellet was resuspended in 550 µl of D_2O (Sigma-Aldrich) containing 0.005% sodium 3-(trimethylsilyl)propionate-2,2,3,3-d4 (TSP) (Sigma-Aldrich) for chemical shift reference. The samples were directly employed for acquisition of 1D ^1H NMR spectra. The spectra were acquired at 700 MHz Bruker Avance spectrometer using standard (Bruker) pulse sequence employing presaturation for suppression of residual water signal. All spectra were acquired at 25 ºC, processed using TopSpin 3.2 (Bruker, USA) and referenced with respect to TSP. Identities of peaks of selected metabolites were assigned by comparison to reference values for chemical shifts (Biological Magnetic Resonance Bank; Ulrich et al. *Nucl. Acids Res*. 2008) and confirmed by titration of the samples with pure compounds (Sigma-Aldrich). Signals corresponding to the glucose, fumarate, and lactate were manually integrated using built-in routines of TopSpin 3.2 software. The signal volumes were normalized to concentration of the TSP employed here as an internal standard. The signals intensities were further normalized to total protein concentration (*vide supra*).

Supplementary Figure 1

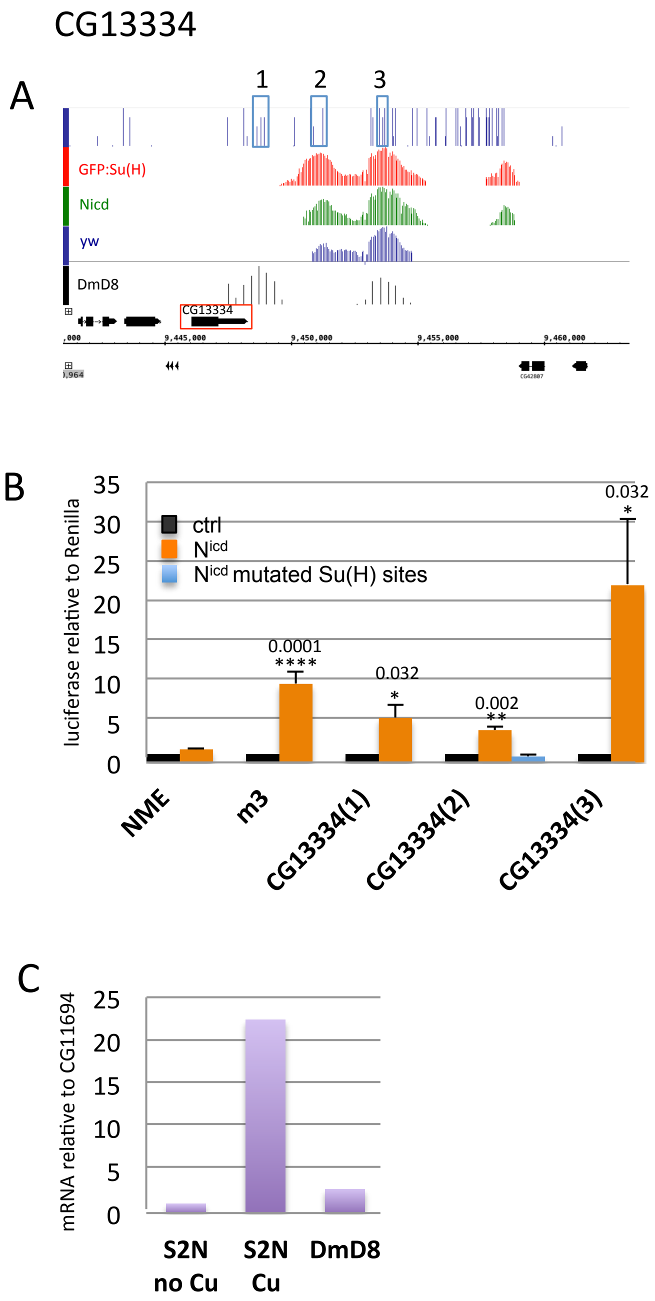

Supplementary Figure 2

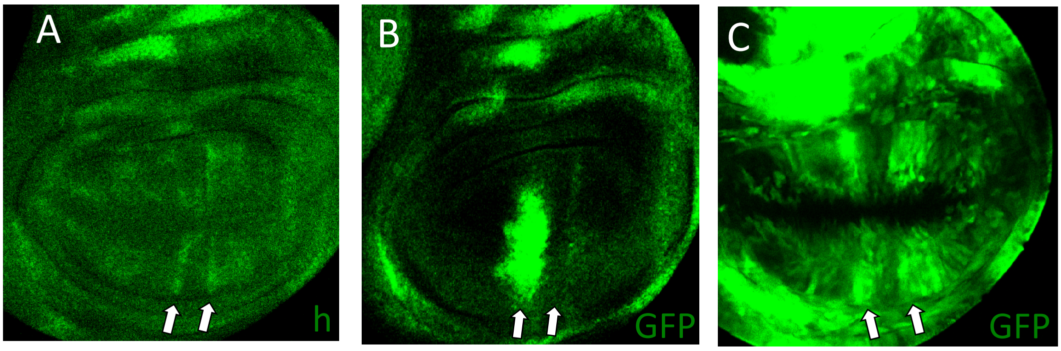

Supplementary Figure 3

A

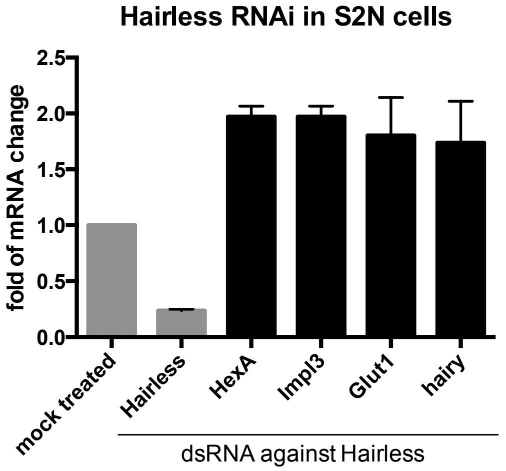

B

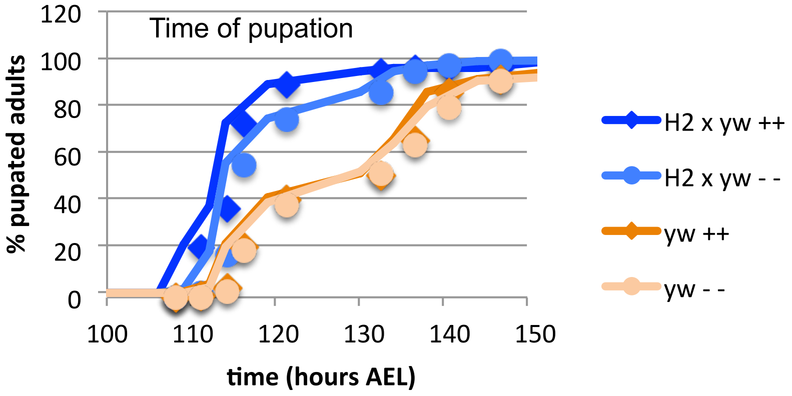

Supplementary Figure 4

A

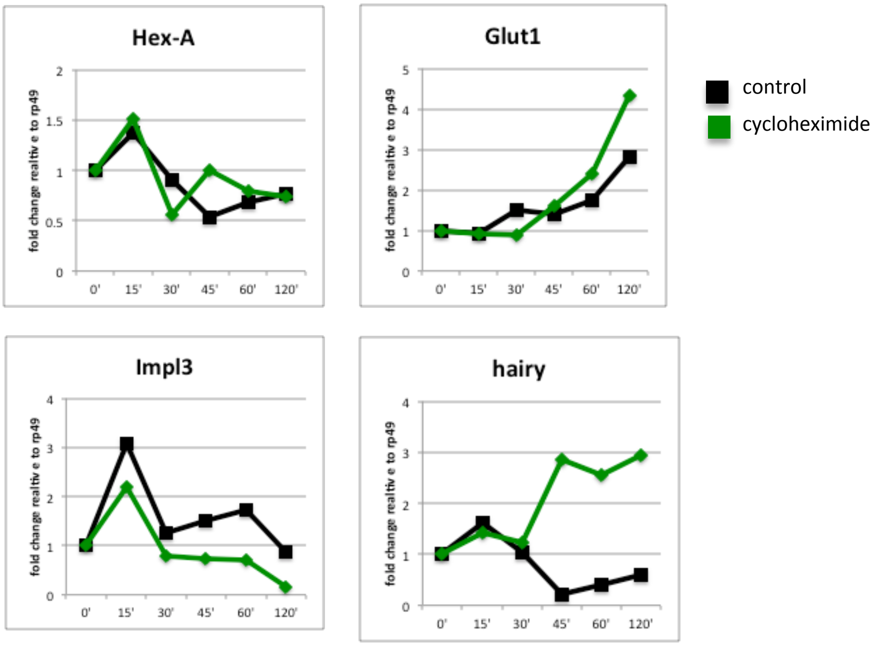

B

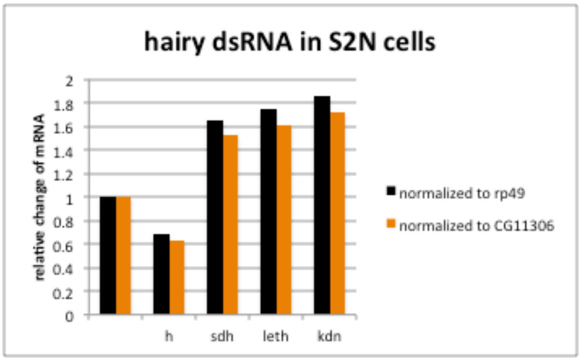

Supplement: Supplementary Material [file rsob150155supp1.pdf]
